# Individual theta-band cortical entrainment to speech in quiet predicts word-in-noise comprehension

**DOI:** 10.1093/texcom/tgad001

**Published:** 2023-01-05

**Authors:** Robert Becker, Alexis Hervais-Adelman

**Affiliations:** Neurolinguistics, Department of Psychology, University of Zurich, Zurich 8050, Switzerland; Neurolinguistics, Department of Psychology, University of Zurich, Zurich 8050, Switzerland; Neuroscience Center Zurich, University of Zurich and Eidgenössische Technische Hochschule Zurich, Zurich 8057, Switzerland

**Keywords:** cerebro-acoustic coherence, functional connectivity, granger causality, speech comprehension, speech-in-noise

## Abstract

Speech elicits brain activity time-locked to its amplitude envelope. The resulting speech-brain synchrony (SBS) is thought to be crucial to speech parsing and comprehension. It has been shown that higher speech-brain coherence is associated with increased speech intelligibility. However, studies depending on the experimental manipulation of speech stimuli do not allow conclusion about the causality of the observed tracking. Here, we investigate whether individual differences in the intrinsic propensity to track the speech envelope when listening to speech-in-quiet is predictive of individual differences in speech-recognition-in-noise, in an independent task. We evaluated the cerebral tracking of speech in source-localized magnetoencephalography, at timescales corresponding to the phrases, words, syllables and phonemes. We found that individual differences in syllabic tracking in right superior temporal gyrus and in left middle temporal gyrus (MTG) were positively associated with recognition accuracy in an independent words-in-noise task. Furthermore, directed connectivity analysis showed that this relationship is partially mediated by top-down connectivity from premotor cortex—associated with speech processing and active sensing in the auditory domain—to left MTG. Thus, the extent of SBS—even during clear speech—reflects an active mechanism of the speech processing system that may confer resilience to noise.

## Introduction

It has been postulated that speech comprehension critically depends upon successful segmentation, or parsing, of the continuous acoustic speech signal into discrete linguistic units (e.g. words, syllables, and phonemes). It is proposed that this is achieved by means of a series of cascaded oscillators that are entrained to the (quasi-)rhythm of the speech signal (Giraud and Poeppel 2012; Peelle and Davis 2012), which is dominated by the syllable rate, which is remarkably consistent across languages (Ding et al. 2017a, 2017b). It is suggested that speech must be parsed into syllable-sized chunks in order to be comprehensible (Ghitza 2011; Ghitza et al. 2012). There is abundant evidence that brain activity does indeed track speech, particularly at the rate of syllables, i.e. in the theta (4–7 Hz) frequency range (Luo et al. 2010).

It has become apparent that this emergent speech-brain synchrony (SBS), which we will further refer to as SBS in short, is unlikely to be simply a passive, bottom-up process, but that it instead reflects an active mechanism or set of mechanisms implicated in speech comprehension. Several findings point to an active role of speech tracking. For example, a rhythmic signal consistent with lexical and syntactic structure building has been shown to emerge during presentation of continuous streams of isochronous monosyllables (Ding et al. 2016, 2017a, 2017b), indicating that the amplitude envelope of brain activity, time-locked to an unfolding speech signal, can reflect higher-order linguistic operations and hierarchical processing in addition to syllable-level processing. Although it has been argued that such a result can equally be explained by a cerebral response that tracks lexico-semantic features of the speech stream rather than hierarchical structure building (Frank and Yang 2018), both positions espouse a perspective whereby the observed tracking, at least to some extent, reflects higher-level, linguistic processes alongside sensory ones (Ding et al. 2016; Frank and Yang 2018; Kaufeld et al. 2020). Another finding is that the strength of SBS has been linked to speech intelligibility (Peelle et al. 2013), suggesting a role in comprehension. Recently, it has also been shown that speech tracking at different time-constants consistent with linguistic decompositions of speech signals—at phrasal, word, syllabic, or phonemic rate—is also differentially perceptually relevant (Keitel et al. 2018). This suggests that viewing at speech envelope activity in different frequency bands—defined by linguistic properties—has its merits as they show that at phrasal and word scale larger speech tracking in left premotor and middle temporal cortex is associated with better comprehension.

The relationship between SBS and speech perception is also a focus in speech tracking studies of older listeners (Decruy et al. 2019; Fuglsang et al. 2020; Goossens et al. 2018; Presacco et al. 2016; Schmitt et al. 2022), in whom there seems to be some consensus that there is relatively greater speech tracking (relative to their younger counterparts), but generally worse speech comprehension performance. Although group level results seem to quite consistently point out this fact, Decruy et al. (2019) show—by looking at the subject-by-subject relationship of speech tracking and comprehension—that larger speech tracking seems indeed to be associated with better speech perception performance, and even more so, for the elderly.

In summary, the notion that SBS, or speech tracking, is associated with speech comprehension is supported by numerous studies. However, as for any empirical correlational study, causality is hard to discern. Most of these studies share an experimental manipulation of intelligibility, for example, by addition of a varying amounts of background noise or by distortion (such as noise vocoding), and examine changes in cerebral speech tracking resulting from the manipulation. Because the manipulation also results in changes to speech intelligibility, it becomes difficult, if not impossible, to distinguish whether the enhanced speech tracking is the cause or consequence of improved intelligibility.

A relatively recent approach to address causality in general in the cognitive neuroscience is the use of noninvasive brain stimulation, such as TMS, tES, or, in an even less invasive vein, M/EEG neurofeedback based on connectivity or other patterns of neuronal activity. Specifically in the field of neurolinguistics, initial studies have already shown success in modulating speech perception or brain responses to speech, using envelope tACS (Riecke et al. 2018; Wilsch et al. 2018; Zoefel et al. 2018; Keshavarzi et al. 2020) or pulsed tES (Ruhnau et al. 2020).

While brain stimulation has the potential to shine a light onto the causal roles of speech tracking it is still dependent on external or extrinsic manipulation, leaving the question of how intrinsic factors may contribute to speech perception largely unanswered. Intrinsic processes and factors in task processing are by now largely accepted as carrying functional meaning beyond simply being noise (Fox et al. 2007; Fox and Raichle 2007) and also, are becoming increasingly studied in speech and language processing (Abrams et al. 2020; Assaneo et al. 2019a, 2019b), this is still an underexplored area worth further investigating. One such candidate of intrinsic variability is the degree of which the brain tracks the rhythm of a speech signal. Under natural conditions, speech tracking is governed by numerous external factors such as signal intelligibility (Peelle et al. 2013), signal-to-noise ratio, and even the linguistic content (Ding et al. 2016). However, even under very well controlled conditions in experimental laboratories, and with identical speech stimuli, individual brains show variable degrees of speech tracking (Keitel et al. 2018). So far it remains unknown whether this is simply neuronal or physiological noise or whether this indicates genuinely important differences in speech processing.

It is possible that, for example, attention may be an intrinsic factor that modulates speech tracking. Up to now, studies investigating the functional implications that can be derived from measurement of single-speaker clear-speech tracking are scarce. In a recent study, Lesenfants and Francart (2020) claimed that speech tracking of clear speech is enhanced by attention. However, it is worth noting, that since they used spectral entropy of the EEG signal as a proxy for attention, rather than a direct behaviorally linked correlate of attention, it is somewhat unclear whether the fluctuations they report in SBS are truly the result of attentional drifts. There are also a number of studies regarding multi-speaker speech tracking and attention, even using clear speech, that have investigated functional role of speech tracking, especially its links to selective attention, e.g. when attending one speaker and ignoring the other (Mesgarani and Chang 2012; Rimmele et al. 2015; Fuglsang et al. 2017; Kerlin et al. 2010; O’Sullivan et al. 2015). However, in such a competitive scenario, blocking and enhancing effects are difficult to disentangle, thus the functional role of speech tracking in this scenario cannot be tell us much about the functional role of speech tracking during listening to noncompetitive, single-speaker speech. Also, in such a scenario it is not clear how much intrinsic variation has contributed to up-modulating speech tracking or whether higher speech tracking and associated better comprehension are actually caused by variations in the concurrent noise level (or other, extrinsic factors).

In the present work, we focus on such inter-individual variability in speech tracking, specifically on the consequences for speech in noise perception. In order to obviate the above-outlined issues of determining causality with respect to brain signatures that may index either the process or consequence of speech comprehension, we exploit a paradigm in which magnetoencephalographic responses of participants subject to the same, continuous speech stimuli are acquired, in the absence of any degradation. Speech in noise recognition performance is then established in a separate task. This approach allows us to directly address the question: how do individual differences in the magnitude of cerebral speech tracking relate to degraded speech comprehension?

Thus, what is still missing so far is an approach that combines analysis of SBS in single-speaker, clear speech with an analysis of whether any observed variation of this type of SBS affects speech comprehension, i.e. has a behavioral impact.

In the present study, we sought to establish whether there are individual differences in the extent of speech-brain entrainment (in the absence of noise) that reflect differences in an independently administered test of speech in noise recognition—a task that calls upon the same auditory and speech processing apparatus, but to a different end. We also ask whether this relationship is specific to certain time scales of the speech input, looking at phrasal, word, syllable, and phoneme rates. The existence of such a relationship—in the presence of a mechanism incompatible with purely sensory-driven processes—would support the proposition that the mechanisms responsible for speech tracking have a significant role in speech processing that manifests as a contribution to speech processing in adverse listening conditions.

## Materials and methods

### Data and participants

We analyzed magnetoencephalography (MEG) data provided by the Human Connectome Project (Larson-Prior et al. 2013; Van Essen et al. 2013). We included data of 53 participants (31 female) who participated in a language task and were native speakers of US English. Participants were on average 29.9 years old (range 22–35 years) and had no psychiatric or neurological conditions and reported no history of hearing impairment. Handedness was assessed using the Edinburgh Handedness questionnaire (Oldfield 1971) and resulted in a mean of 69.72 and a range of −60 to 100 (with positive numbers indicating more right-handedness, total range of −100 to 100).

### Experimental design of listening task and word-in-noise task

#### MEG data, listening task

The language task is an active listening task in quiet (i.e. noise-free), consisting of 14 English short auditory stories, originally adapted from Aesop’s fables, narrated by a male speaker in English. The experiment was split into 2 sessions, with 7 fables presented in each (mean duration 20.3 s, range 13.7—24 s). After each story, participants answered a comprehension question. All participants listened to the 14 stories in the same order.

#### Determining frequencies of phrases, words, syllables, and phonemes

Following a procedure employed in an earlier study of SBS by Keitel et al. (2018), transcripts of the speech materials were tagged using forced alignment using the MAUS system (Schiel 1997) and manually corrected. The boundaries of phrases (elements demarcated with a relevant punctuation mark: stop, comma, dash or hyphen, semi-colon, colon), words, syllables, and phonemes were marked and the maximum and minimum rate of occurrence of these was established over the 14 fables individually. Analogous to Keitel et al. (2018), the maxima and minima across all stories were used to define a pass-band edges for filtering both the speech and the MEG signal to analyze the coherence of the 2 time series. Signals were filtered using a 4th order, zero-phase Butterworth filter implemented in MATLAB (The Mathworks, Natick, USA). The following frequencies were estimated: phrasal rate in this corpus was 0.2–2 Hz, word rate 2.4–4.9 Hz, syllable rate ranged from 3.4 to 6.5 Hz (there is some overlap between the 2 rates), and phoneme rate from 8.4 to 14.1 Hz.

#### Modulation spectrum

**Fig. 1:**
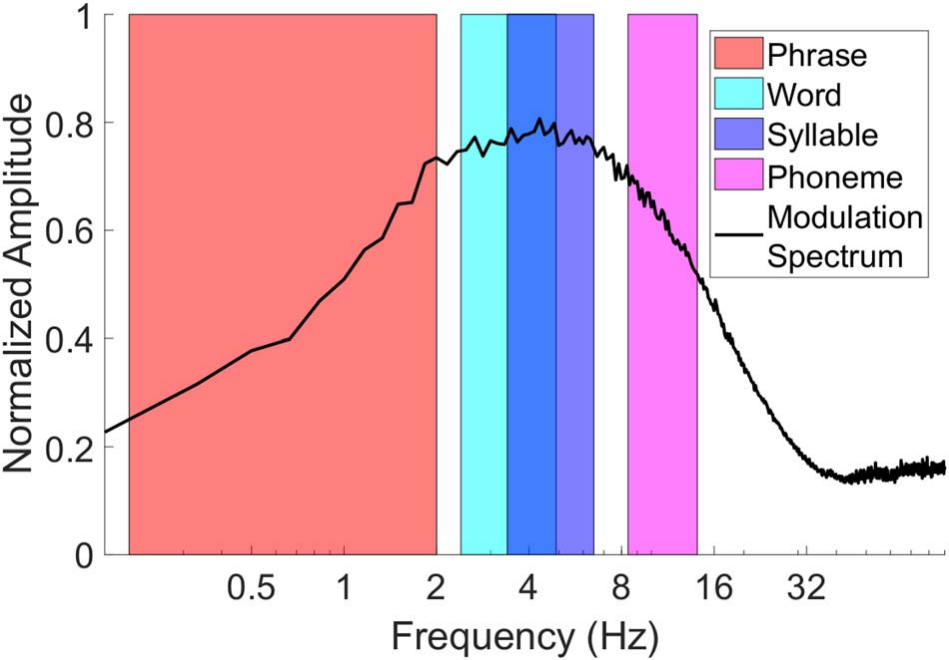
Average modulation spectrum and spectral ranges of stimulus material (Aesop’s fables) used in this study. Before averaging across stories, the normalized modulation spectrum for each short story was calculated as in Ding et al. (2017a, 2017b), bands were derived as in Keitel et al. (2018), spectral ranges are indicated by the colored fill-ins. NB: There is overlap between word and syllable range (as indicated by the blueish color shade).

We computed the modulation spectrum of the speech material (Fig. 1), following the approach of Ding et al. (2017a, 2017b).

#### Behavioral data

##### Word-in-noise recognition

**Fig. 2:**
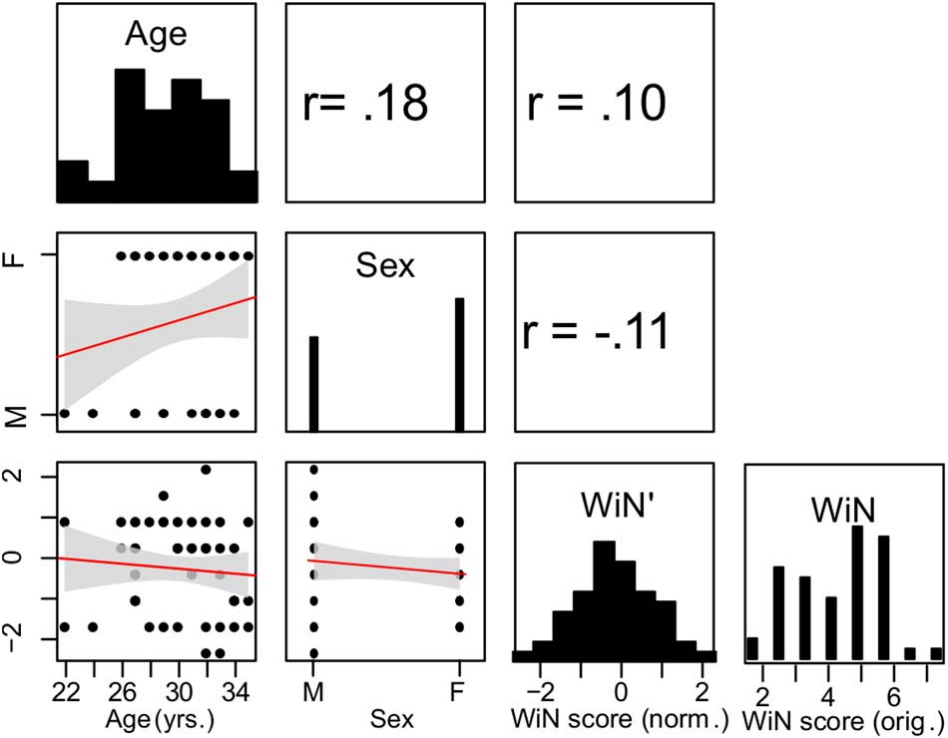
Descriptive overview of demographics of population and distribution of the behavioral-cognitive variables used in this study. This matrix shows age, gender, and the participants’ scores for WiN recognition. The diagonal shows the distribution of individual values for the population characteristics and the transformed WiN measures (denoted WiN’, see Materials and Methods for details of transformation). Original WiN scores are shown in the last column. Correlations (linear Pearson’s and point-biserial correlation) and scatter plots are derived from the transformed version of WiN. Linear fits are shown in red, with confidence intervals (±95%) shown in gray. Units of the original measures: WiN—in dB SNR. Transformed data (WiN’) are Gaussianized by rank-based inverse normalization and also normalized to zero-mean and unit variance (see Materials and Methods). No significant correlations were observed.

The same group of participants was tested for their word in noise (WiN) recognition performance using the WiN test from the NIH Test Battery. This test involves the recognition of monosyllabic words embedded in multi-talker babble. Participants hear a list of 5 words at progressively decreasing levels of SNR (decreasing in 4 dB increments, ranging from 26 to −2 dB minimum SNR). Correct responses are collected and the protocol is stopped when a participant cannot correctly identify any word of the list. Then using the formula: 50% sNR threshold = 26 dB − raw score ^*^ 0.8, the raw score (number of correct responses) is converted to a 50% correct dB SNR threshold (Wilson et al. 2005, 2007). Mean performance was 4.6 dB SNR (range: 2–7.6 dB) for the computed 50% SNR threshold. A visualization of WiN scores across participants and other demographics is given in Fig. 2.

#### MEG data acquisition

MEG data were acquired with a 4D BTI MEG device with 248 magnetometer sensors covering the whole head. The data were preprocessed according to the HCP protocol—cleaned of eye and heart artifacts by independent component analysis, notch filtered and bad time-periods in any trials were masked out.

#### Source reconstruction

Following an approach used in a previous study (Houweling et al. 2020), data were down-sampled to 200 Hz, filtered between 1 and 48 Hz, and then sources reconstructed by employing linearly constrained minimum variance beamforming, using T1-weighted MR individual head images for reconstruction and alignment. Resulting source space is in an 8 × 8 × 8 mm resolution grid, containing 5,798 in-brain source voxels.

#### Evaluation of SBS

The extent and the fidelity of speech tracking were estimated by Gaussian-copula mutual information (GCMI, a procedure described in full by Ince et al. 2017). This measure is an index of SBS. The approach used here is based on information theory (Shannon 1948) and measures the quantity of information transmitted between the speech signal and the concurrent brain signal. This extension on the concept of mutual information allows it to be run on continuous (speech and brain) data with robust and interpretable estimation of is the strength of communication or amount of transmission of information (in bits). Expecting an average delay regarding the trailing of cortical processing with respect to the input, of ~120 ms, we chose to shift the brain response to speech input by that amount, thus defining the base delay between speech and brain response for the following SBS analysis, consistent with previous studies (e.g. Keitel et al. 2018; Lesenfants and Francart 2020). Any additional deviation from that base delay (i.e. more or less delay) was accounted for by the bivariate implementation of the GCMI approach that models the brain signal as 2 time series, which are essentially the real and the imaginary part of the same (complex valued) Hilbert-transformed bandpass filtered time series, resulting in the original plus its 90-degree shifted variant. This allows a range of delays to be accounted for, e.g. in the case of theta band activity with a base frequency of 4 Hz, an approximate range of 80–180 ms of delay (120 ms ± a shift of 90°, or a quarter phase) would be covered.

In order to derive significance of GCMI, permutation testing was employed, as per Keitel et al. (2018). First, surrogate speech tracking data were generated, i.e. GCMI was computed for the MEG recordings to mismatched speech (for a total of 50 randomizations, swapping stories across trials). In case of a mismatch of length, recordings were trimmed accordingly. At each voxel and subject, these 50 runs were then averaged to result in one, individual surrogate GCMI per voxel and frequency band (phrase, word, syllable, phoneme). Then, at each voxel, a paired *t*-test was performed (across subjects), contrasting real and surrogate GCMI values. This resulted in one *t*-value per voxel (and frequency band). After identifying the critical *t*-value at *P* ≤ 0.001, and thresholding the data accordingly, remaining clusters were examined and quantified by summing up their respective *t*-values. At this point, permutation statistics were performed by swapping subject labels (taking into account genetic relationship of subjects to each other - the HCP data set contains several subjects who are related, including siblings and dizygotic and monozygotic twins) and repeating the above-described analysis 1,000 times. At each of these runs, thresholding was performed as in the unpermuted data and cluster-based maximum statistics were collected (summing up *t*-values per supra-threshold cluster and storing the top three largest clusters per run). Any clusters exceeding the 95th percentile of the null distribution (for the first to third largest clusters and corresponding test statistics) were considered significant, following Maris and Oostenveld (2007).

### Relationship between SBS and WiN

Relationships between SBS (as estimated by mutual information) and WiN were evaluated using linear correlation. To ensure normal distribution, rank-based inverse normal transformation was applied to the original raw WiN scores. Missing values of WiN (one participant) were imputed. Also, WiN and SBS were deconfounded for age, since we wished to exclude any age-related effects (despite the relatively narrow age range of the participants) by regressing out in both variables the participants age (in years). Correlation analysis was executed in source space between all source voxels exhibiting significant SBS and WiN. Permutation statistics were used, establishing null models by swapping subject labels and performing identical analyses 1,000 times. This established a null distribution of correlation coefficients. Any source voxel with correlation coefficient exceeding the top 95th percentile was considered significant. In order to take care of spurious effects, 33 single voxels or clusters consisting of fewer than 5 contiguous voxels were discarded. By doing so—out of a total of 3,724 univariate voxelwise correlations across all band-specific ROIs—we removed 0.89% of all correlations performed, representing a relatively conservative level of discarding spurious or false positive results.

#### Directed connectivity

One important consideration in interpreting any potential relation between SBS in clear speech and WiN may be the contribution of peripheral auditory perceptual factors, such as individuals’ hearing level. These may play a causal role in any observed effects. While, in this study, we (i) focus on younger listeners who self-reported as having no history of hearing impairment and (ii) examine the relationship between their amount of SBS expressed during a speech comprehension task (during ongoing MEG recording) and their WiN recognition ability assessed in an independent task, we are acutely aware of the possibility that individual differences in SBS to clear speech may arise from individual differences in the auditory periphery, e.g. different auditory sensitivity or undetected hearing impairment. In order to help corroborate that any relationship between SBS and task processing is because of central processes that contribute to individual differences in speech tracking mechanisms, we therefore also investigated the role of top-down (TD) connectivity in generating SBS and in mediating any observable SBS–WiN relationship. This would make purely bottom-up-driven processes (e.g. auditory thresholds or features of the auditory brainstem) less likely as main factors and justify the inference of some degree of central causality. To this end, we assessed directed connectivity between a set of fronto-temporal brain areas associated with language speech processing following similar approaches to Schoffelen et al. (2017) and Arana et al. (2020), where the temporal target areas in each hemisphere were the areas showing significant SBS–WiN relationship. This was our first set of connections modeled. In a second set of connections, we modeled connections between the areas showing significant SBS–WiN relationships (SBS-ROIs) and left and right primary auditory cortex. We then compared the contributions of families of TD and bottom-up connections to SBS and WiN. The parcellation we used was derived from the Brainnetome atlas (Fan et al. 2016), resulting initially in as set of 52 language-related parcels.

At the original spatial resolution of the atlas (often used for fMRI investigations), the resulting correlation matrix (i.e. correlations across parcel time series) was often rank deficient causing the subsequent source leakage correction to fail. Therefore, we reduced the number of parcels by merging those neighboring parcels that were most strongly correlated to each other, using k-means clustering (with *k* = 30), such that collinearity was avoided. In order to establish potential relationships between connectivity and SBS, in this parcellation, we also included parcels defined by their significant SBS–WiN relationship (at syllable level), which were also comparable in size to the other parcels used in the parcellation schema (these parcels are further called SBS-ROIs). These ROIs resulted in two parcels, residing in left and right temporary gyrus, with a right parcel centered in superior temporal gyrus (STG), whereas the left parcel was centered in middle temporal gyrus (MTG).

Subsequently, parcel time courses were extracted by principal component analysis (PCA) and the first principal components (PCs) of each parcel were subjected to multivariate source leakage correction as described by Colclough et al. (2015).

### Fronto-temporal connectivity during speech processing in quiet

We modeled the following four sets of connections: first, TD connectivity from frontal areas to SBS-ROIs (right STG/left MTG), i.e. the areas that showed both SBS and SBS–WiN relationship. This resulted in a set of 10 (5 × 2 hemispheres) left and right frontal parcels sending TD information to SBS-ROI areas.

**Fig. 3:**
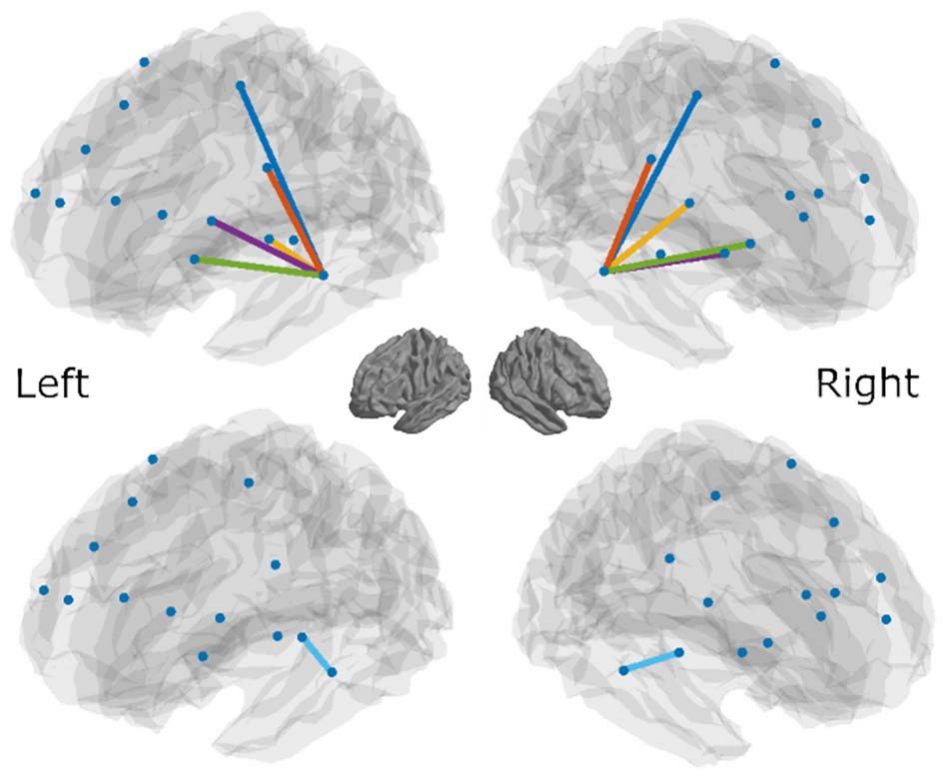
Visualization of the edges (connections) used for granger causality and subsequent analyses. Top: 1 network consists of 5 parcels for each hemisphere, comprising frontal areas (superior, inferior frontal gyrus and PRG regions) involved in language processing, connected to down-stream regions of interest that are defined by significant SBS (termed SBS-ROIs), centered in left MTG and right STG. Bottom: a second network includes 2 parcels centered in left and right primary auditory cortex and their connections to the two SBS-ROIs.

Second, we modeled the inverted connections, that is bottom-up from right STG, and left MTG, to frontal areas. Third, we modeled bottom-up connectivity from left and right primary auditory cortex to ipsilateral SBS-ROIs (STG and MTG, respectively), and fourth, we model the TD influence of left/right S/MTG on ipsilateral primary auditory cortices. This resulted in a total of 24 modeled connections (10 TD fronto-temporal connections plus their bottom-up counterpart and 2 bottom-up connections from primary auditory cortex to SBS-ROIs and vice versa). For the specific nodes selected and their anatomical labels please see Supplementary Table 1 and Fig. 3 for a visualization of the resulting networks. After selecting these edges, spectrally resolved Granger causality was computed for each subject and at each edge, with frequency steps of 1–100 Hz, the Nyquist frequency.

#### Relationships between directed connectivity and WiN and SBS

After having computed spectrally resolved connectivity measures, we tested whether these explain inter-individual differences in SBS and speech in noise comprehension (WiN). We aimed to discover whether TD connections modulate WiN, SBS, or both. Since we are relating one set of multidimensional variables—connectivity—to another set of multiple variables—WiN and SBS—canonical correlation analysis (CCA) is a natural methodological choice (see for example Smith et al. 2015; Becker and Hervais-Adelman 2020).

For each of the 4 defined TD and bottom-up networks (i.e. the frontal-to-SBS-ROIs and vice versa for each hemisphere, plus primary-auditory-cortex-to-SBS-ROIs and vice versa), directed network connectivity was initially represented by a matrix of either 53 participants × 5 edges × 100 frequency steps (for the frontal-to-SBS-ROI and vice versa networks) or by a matrix of 53 × 2 × 100 for the primary-auditory-to-SBS-ROI (and vice versa) networks. Because of the high dimensionality of this data in comparison to the moderate sample size, we performed an initial dimensionality reduction. This was achieved using PCA of the spectrally resolved data for each node, retaining 3 PCs out of 100 frequency bins, explaining a minimum of 98% variance. These 3 components per node, per participant compose 1 dimension-reduced set of connectivity variables (now being 53 × 5 × 3 for the fronto-temporal model and 53 × 2 × 3 for the primary-auditory-to-SBS-ROI model) fed into the CCA. The other set of variables consisted of SBS scores (from the SBS-ROIs, extracted at syllable rate) and the scores from the WiN task, being normalized to zero-mean unit variance before performing CCA. The CCA, testing the relationship between these two sets of variables—i.e. the spectrally resolved connectivity on the one hand and SBS and WiN on the other—was performed per edge and hemisphere. SBS and WiN variables were normalized to zero-mean unit variance. For each run of the CCA, permutation testing (swapping the subject labels, *n* = 1,000) was executed to establish significance of the canonical mode linking connectivity and WiN/SBS. To ensure statistical robustness, *P*-values resulting from all tests here (*n* = 24, which include the 5 edges on each hemisphere tested for both TD and bottom-up effects of the network involving frontal and SBS-ROI areas, and the 2 edges on each hemisphere tested for TD and bottom-up effects in the network involving primary auditory cortex and the SBS-ROI areas) were subjected to a correction for multiple comparisons using the false discovery rate method (Benjamini and Hochberg 1995). After this correction, in case of a significant canonical mode—which is primarily a set of linear weights for both the connectivity set and the WiN/SBS set that obtain the best possible match between the two sets of variables—post-hoc linear correlation analyses are performed, between the CCA-weighted connectivity patterns and WiN and SBS variables separately, to further characterize and enable interpretation of the observed canonical mode.

#### Mediation analysis

After using CCA to identify edges that might be linked to SBS or WiN performance, we investigated whether TD connectivity had a mediating effect on the SBS–WiN relationship. The existence of such a mediation, even if partial, would rule out the possibility that an SBS–WiN relationship is driven purely by sensory, peripheral factors. Mediation analysis was carried out using MeMoBootR (Buchanan 2018) in R (R Core Team 2021). We included SBS values (from the SBS-ROI exhibiting significant speech tracking and also a significant SBS–WiN relationship, again at syllable rate), WiN scores for the participants and the aggregate TD connectivity measure calculated as the Granger Causality estimates for TD edges weighted by the CCA coefficients from the previous CCA analysis, representing the first canonical mode, linking connectivity to SBS and WiN.

To test whether mediation was full or partial, we conducted two tests: Sobel’s *z* test and complementary to this, bootstrap confidence interval estimation, which is sometimes viewed as a more powerful alternative (Zhao et al. 2010). There, instead of potentially incorrectly assuming a normal distribution, an empirical sampling distribution of *a* × *b* (the indirect path) is generated instead (Preacher and Hayes 2004, 2008). The bootstrap confidence interval was estimated on the basis of 1,000 replicates.

## Results

### Speech-brain synchrony

**Fig. 4:**
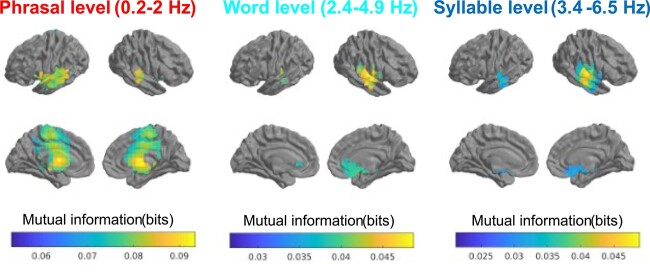
Significant SBS (*P* < 0.05), as measured by GCMI, observed at 3 different frequency bands. At phrasal rate, left temporal areas show stronger SBS than corresponding right areas and there are also extensive subcortical/thalamic foci, whereas at faster rates, i.e. word and syllable rate, the focus of SBS shifts to right temporal areas including primary auditory cortex. Please note that mutual information is not frequency independent, and tends to become larger with decreasing frequency, as indicated by differences in scale.

In source space, significant SBS (as estimated by mutual information here and throughout this work) was found at phrasal (0.2–2 Hz), word (2.4–4.9 Hz), and syllable rate (3.4–6.5 Hz), but not at the phoneme rate (8.4–14.1 Hz). Results are visualized in Fig. 4. We will now outline the findings ordered by frequency band, from largest to smallest linguistic unit:

Phrasal rate. Using cluster-based maximum test statistics (where we compare max statistics for the first, second, and third biggest cluster, denoted *t*_sum1st_, *t*_sum2nd_, and *t*_sum3rd_, respectively), we found one extensive, cross-hemispheric, slightly left-centered cluster at phrasal rate (*t*_sum1st_ = 1,965, *P* < 0.001, 403 voxels, MNI center coordinates [−20, −12, 15]). Word rate. At word rate, we found 3 significant clusters, 1 centered in right primary auditory cortex (*t*_sum1st_ = 491.81, *P* < 0.001, 109 voxels, MNI [54, −16, 4]),1 one in left hippocampus (*t*_sum2nd_ = 297.25, *P* < 0.001, 64 voxels, MNI [8, 15, −26]), and 1 in left primary auditory cortex (*t*_sum3rd_ = 64.64, *P* < 0.001, 15 voxels, MNI [−48, −26, −2]). Syllable rate. At syllable rate, we found 2 significant clusters, 1 in right primary auditory cortex (*t*_sum1st_ = 997.43, *P* < 0.001, 193 voxels, centered at [56, −15, 0]) and 1 in left medial temporal gyrus (*t*_sum3rd_ = 167.62, *P* < 0.001, 37 voxels centered at [−61, −22, −7]). These are based in right primary auditory cortex and left medial temporal gyrus.

Thus, from the slowest, phrasal, rate to faster rates, there was a transition of left to right-dominant focus in temporal cortex.

### WiN–SBS relationship

In order to establish whether individual differences in SBS account for variation in behavior in an independent WiN recognition task, and where, we analyzed the relationship between SBS (as measured by GCMI) and WiN, in a region of interest defined by significant SBS. We note that while the words-in-noise test is clearly based on words and hence emphasize word-level processing, these words are monosyllabic and furthermore embedded in multi-talker babble. Therefore, predictions regarding the most relevant linguistic rates are not straightforward—it is possible that inter-individual differences in the ability to suppress noise may manifest in different linguistic rates. Thus, in this work, we chose to include all linguistic rates for investigating a potential relationship between speech tracking (or SBS) and WiN.

**Fig. 5:**
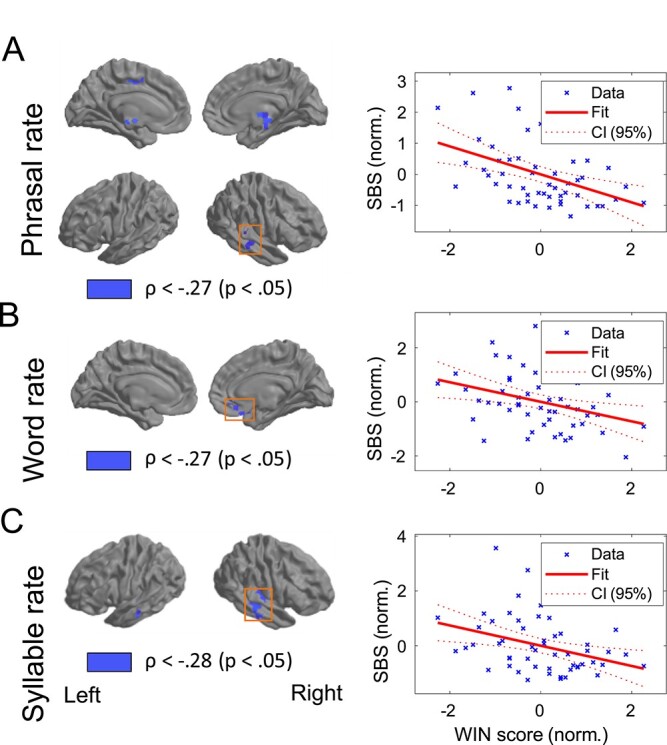
Visualizing the relationship of SBS vs WiN, showing the brain areas with significant relationship between speech tracking and WiN comprehension, across all rates that showed a significant relationship (and thresholded at *P* < 0.05). Both at phrasal, word and syllable rate, there was a negative correlation between speech tracking and WiN comprehension. At phrasal rate (A), bilateral subthalamic areas, left ventral anterior cingulate and right STG were observed. At word rate (B), medial orbitofrontal areas were showing significant SBS, whereas at syllable rate (C), SBS in a right cluster in STG was significant. Scatter plots on the right illustrate the negative correlation between SBS and WiN at the different rates, all indicating better recognition performance (= lower WiN score) with higher speech tracking in quiet. At each rate, the chosen cluster is indicated by an orange-lined box.

Results ordered by frequency bands (linguistic rates) are as follows (presented in Fig. 5): Phrasal rate. At *phrasal rate*, we found 3 clusters of significant WiN–SBS relationship (all negative): 1 subcortical (6 voxels, in a medial right subthalamic area [3, −15, −7], mean *ρ* = −0.32), 1 right STG (13 voxels [54, −28, 1], mean *ρ* = −0.36), and 1 in left ventral anterior cingulate (6 voxels [−12, −9, 45], mean *ρ* = −0.32). Word rate. At *word rate*, we found a significant relationship of WiN performance and SBS at one right orbitofrontal cluster (15 voxels [9, 27, −28], mean *ρ* = −.33).

Syllable rate*.* At *syllable rate*, we found a significant and negative relationship between WiN and SBS, in a cluster centered on right STG (47 voxels [58, −22, −7], mean *ρ* = −0.31), and a smaller left MTG cluster (6 voxels [−61, −17, −8], mean *ρ* = −0.32, see Fig. 5 for visualization and scatterplot for the relationship of SBS and WiN in the right temporal cluster). Phoneme rate. At *the phoneme rate*, there was no ROI with significant SBS, so this relationship was not tested.

Notably, within the other 3 bands, and within the defined SBS-ROIs, there were exclusively voxels showing a negative relationship between SBS and WiN, meaning higher speech tracking (as estimated by mutual information) was associated with higher speech comprehension across individuals. In terms of numbers of voxels with significant SBS–WiN relationship, syllable rate SBS had the largest number of voxels linked to WiN (with a total of 53 voxels), followed by phrasal rate SBS–WiN (with a total of 25 voxels) and word rate SBS–WiN (with 15 voxels).

### Relationship between directed connectivity and WiN/SBS. Frontal to SBS-ROIs—TD and bottom-up network connectivity

**Fig. 6:**
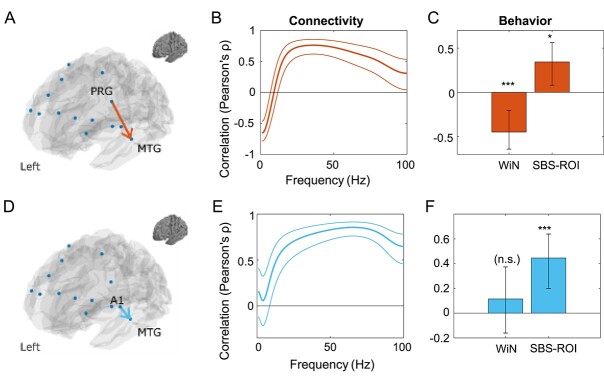
CCA of the relationship between TD/bottom-up connectivity and SBS and WiN yields 2 canonical modes with significant relationships. (A–C) For TD connectivity from frontal to SBS-ROIs (A), CCA yielded 1 significant mode in a left-hemisphere parcel. Its spectrally resolved connectivity profile is composed of beta and lower gamma activity (B) upmodulating SBS and down-modulating WiN scores (= positive impact on WiN performance), whereas lower-frequency (alpha and below) activity shows inverse relationship to SBS and WiN (= negative impact), see c. (D–F) For connectivity between primary auditory cortex and SBS-ROIs (left MTG and right STG), we observed 1 canonical mode, from left primary auditory cortex to left MTG SBS-ROI (D), with a spectral profile showing mostly activity in the gamma range (E). This mode is significantly up-modulating SBS, however was not significantly linked to WiN (F). Abbreviation: A1, primary auditory cortex. Asterisks indicate significance of post hoc single-variable correlations (^*^ = *P* < 0.05, ^*^^*^ = *P* < 0.01, ^*^^*^^*^ = *P* < 0.001). For connectivity (B, E), thick lines indicate correlation coefficients with the neighboring thin lines surrounding it indicating the 95% confidence interval. Analogously, the bar plots for WiN and SBS (C, F) are complemented by 95% confidence intervals as well in the form of error bars.

For the set of TD connections with frontal and prefrontal starting points terminating in corresponding left and right SBS-ROIs (extracted from left medial and right STG exhibiting significant SBS at syllable rate), resulting in 5 nodes and edges per hemisphere (see Fig. 3, top row, and Supplementary Table 1 for information on localization and anatomical characterization), we found one canonical mode with a significant link between left-hemisphere TD connectivity and SBS and WiN (*ρ*_tdconn_ = 0.50, *P* = 0.031, corr., see Fig. 6), specifically a left precentral gyrus (PRG) parcel, encompassing parts of Brodmann areas 4 and 6 (centered at MNI x,z,y: −49, −2, 33) connected to the left SBS-ROI. Resulting canonical coefficients of SBS and WiN for this mode were the same order of magnitude (−0.45 for SBS, 0.76 for WiN), indicating that this mode does not weight one variable substantially more strongly than the other. Since significance of a CCA mode per se is somewhat abstract, a usual approach involves correlating the mode (the canonical scores) with either set of variables, which further characterizes the observed canonical mode.

For example, post-hoc correlations of the two variables SBS and WiN with the observed mode (the canonical scores) yielded significant relationships for both (SBS: *ρ* = −0.45, *P* < 0.001, WiN: *ρ* = 0.34, *P* = 0.012, see Fig. 6C). Directed connectivity as measured here using Granger causality was executed across the entire available frequency bands, thus the mode itself encompasses all the different linguistic rates (phrasal, word, syllable, or phoneme) and in fact goes beyond those rates. SBS on the other hand by definition is derived from a specific linguistic rate and here, syllable rate was chosen. At this rate, we found the largest clusters with significant SBS–WiN relationship, with sites according to medial/superior temporal gyri, and also in both hemispheres. Thus, when referring to SBS-ROIs (left and right) for following analyses we refer to syllable rate SBS at the ROIS identified in Fig. 5.

Again, to gain insight into the otherwise abstract nature of the canonical mode, now the canonical scores (of the first, significant mode) were correlated with the fully resolved connectivity profile for each edge, providing a more interpretable picture regarding the involved spectral connectivity (see Fig. 6B, top panel). Beyond simply establishing that there exist spectral connectivity components significantly linked to speech tracking and/or behavior, this characterizes which part of the spectrally resolved connectivity is relevant with regard to the canonical mode. The results show that lower-frequency connectivity (below the alpha frequency band, i.e. <10 Hz) has a negative impact on the both SBS and WiN, whereas faster, i.e. beta and low-gamma connectivity (>20 Hz) has a strong positive correlation with the two variables, indicating stronger speech tracking and better WiN performance for stronger beta and low-gamma connectivity. In contrast, WiN and strength of *bottom-up connectivity* between SBS-ROIs and frontal areas was not significantly correlated in any of the fronto-temporal edges (with strongest correlation being *ρ* < 0.41, *P* = 0.25).

### Primary auditory cortext to SBS-ROIs—TD and bottom-up network connectivity

Testing the relationship of the TD and bottom-up network between primary auditory cortex and the SBS-ROIs also yielded one significant mode, which linked the bottom-up connectivity from primary auditory cortex to SBS-ROIs to SBS/WiN variables (ρ = 0.52, *P* = 0.02, corrected for multiple comparisons, Fig. 6D–F). This was also exclusively observed in the left hemisphere. Resulting canonical coefficients of SBS and WiN for this mode were −1.03 for SBS, and −0.55 for WiN. Post-hoc testing of the link of this mode to SBS and WiN separately yielded a significant, positive correlation only for bottom-up connectivity and SBS (*ρ* = 0.45, *P* < 0.001), but not for WiN (*ρ* = 0.12, *P* = 0.41), implying that in this bottom-up connection, stronger connectivity was linked to stronger speech tracking (Fig. 6F).

### TD connectivity mediates relationship between SBS and WiN

After having identified a significant relationship between SBS and WiN, and a TD connection being linked to both SBS and WiN, we employed mediation analysis to determine whether TD connectivity identified by CCA acts as a mediator for the effect of SBS (the predictor) on WiN (the outcome). Resulting path coefficients were as follows: path a, i.e. SBS on TD = 32.72 (SE 12.50, *P* = 0.012), path b, i.e. TD on WiN = −0.38 (SE 0.13, *P* = 0.005). Path c, i.e. total effect c or effect of SBS on WiN, absent any mediation = −29.94 (SE 12.38, *P* = 0.019). The direct effect as reflected by path coefficient c’, i.e. including TD as mediator = −17.59 (SE 12.31, *P* = 0.159), respectively. The indirect effect, as computed by the difference method, c − c’, was −12.35 (see Supplementary Fig. 1 for a graphical summary of this mediation analysis). Sobel test for significance of the mediation showed a trend, failing to reach significance (*z*-score = −1.89, *P* = 0.059). A complementary approach to estimate the significance of the mediation, using bootstrapping (Preacher and Hayes 2004, 2008), yielded a 95% confidence interval of [−22.90, −0.89] being outside 0, and thus indicating a significant result. This is taken as a sign of a partial mediation, here, specifically of the effect of SBS on WiN by TD connectivity from a left PRG cluster.

## Discussion

We find that auditory-cortical speech tracking in quiet predicts noisy speech recognition in an independent behavioral task. Furthermore, the relationship between speech tracking at the syllable rate and noisy speech recognition was mediated by left-hemispheric fronto-temporal connectivity. The analyses presented above suggest that the individual tendency to entrain to the syllabic rhythm of speech is reflective of the efficacy of an active perceptual mechanism that contributes to the robustness of speech-in-noise perception.

The largest cluster of source voxels linking SBS to WiN performance is located at the right STG, and is observed at the syllable rate. Given that the words used in the WiN test are exclusively monosyllabic (Wilson et al. 2005, 2007), and that there was substantial overlap in the frequency bands assigned to words and syllables in the speech stimuli analyzed, it is not surprising that indeed syllable rate SBS is more predictive of WiN performance than word rate SBS—which includes multisyllabic words as well.

Apart from the fact that syllable rate shows the most extensive clusters linking SBS and WiN, we also observe a significant relationship between SBS and WiN in temporal cortices of *both* hemispheres, in right STG and left MTG at syllable rate. While the right-hemispheric cluster is larger, the left-hemispheric cluster is the one that appears more amenable to TD influences relevant to ongoing speech processing and independent WiN recognition performance—it is there that SBS is positively related to TD connectivity and the same TD connectivity targeting this cluster is also linked to WiN, mediating the relationship between the two. This is not the case for the right hemisphere cluster. Whether this reflects more “automatic” or passive speech tracking on the right is unclear, but this hemispheric distinction merits further investigation.

The fact that—independent of the examined linguistic rate—we exclusively found negative correlations of SBS and WiN implies that greater speech tracking in clear speech is consistently indicative of greater robustness of speech perception in the face of masking noise. This relationship has been reported previously for experimentally manipulated speech, either at the group (e.g. Peelle et al. 2013; Keitel et al. 2018) or individual level (Decruy et al. 2019). This may imply that despite the methodological difference between our study and others, similar processes may be at play, no matter whether speech perception is effortful or not, which speaks to the universal role of speech tracking irrespective of listening conditions.

The observation of neuroanatomical loci such as superior or medial temporal gyri in the context of (syllable rate) speech tracking and its interpretation is relatively straightforward and plausible—these are higher-level, speech-processing areas that probably receive some version or aspect of the processed speech stream from the lower-level, primary auditory cortices. The additional fact that both left and right higher-level temporal areas show a link between speech tracking and the ability to report words in noise is also intuitive and makes the syllable rate findings maybe the most robust or most impactful findings across all the rates we looked at. This does not invalidate any of the other bands—i.e. the phrasal and word rate—and their results. However, while there is evidence for both thalamic and subthalamic involvement in semantic and syntactic processing (Wahl et al. 2008) and evidence for a role of anterior cingulate in contextual processing of speech (Smirnov et al. 2014), the exact functional interpretation of these areas linking phrasal or word rate SBS and monosyllabic WiN performance may need further investigation in order to gain a better understanding of the underlying mechanisms.

### Inter-individual differences in SBS in quiet index individual differences in speech-in-noise processing

The significant SBS–WiN relationship reported above is consistent with existing results showing that SBS predicts stimulus intelligibility (Peelle et al. 2013; Fuglsang et al. 2017; Keitel et al. 2018; Decruy et al. 2019). However, as results that relate ongoing SBS to ongoing speech comprehension suffer from an inherent circularity—does SBS drive comprehension, or does it result from it, as would be predicted by accounts of SBS that emphasize the role of higher-level linguistic information in generating speech-signal locked oscillations (e.g. Ding et al. 2016; Frank and Yang 2018; Kaufeld et al. 2020)? The results reported here are free of such interpretational difficulties—SBS in quiet predicts WiN recognition ability in an independent task. Thus, the degree of speech-brain entrainment (at the syllable level) that an individual tends to exhibit *to speech in quiet* is an index of a property of their speech processing system that confers resilience to noise (in the context of a single word recognition task). Although we cannot conclude from the present data that this mechanism directly influences WiN recognition, we argue that the results we observe are further evidence for an active and functionally relevant role for syllabic entrainment in speech perception, which can particularly benefit speech perception in adverse listening conditions. This is further corroborated by the results of our connectivity-based analyses. First, we saw that there is TD connectivity that is significantly related to WIN, whereas bottom-up connectivity did not exhibit such a relationship. Second, regarding the putative impact of SBS on WiN, we found that at least part of this relationship was explained by inter-individual differences in beta and gamma connectivity in a fronto-temporal network, and that TD influences can modify speech tracking has been shown before, but often in the context of multi-speaker situations (Fuglsang et al. 2017) or as recently, in a study by Lesenfants and Francart (2020), where putative attentional processes exerted such influence, even during processing of clear speech. This may be relevant to our findings allowing the interpretation that there are TD mechanisms at work shaping the speech tracking response actively, more than what would be expected from a passive purely bottom-up mechanism.

### Implications for the role of SBS in speech processing

The findings indicate a likely contribution of TD mechanisms and thus, an active process. This might be related to active inference, one of the core principles of predictive coding (Friston and Kiebel 2009), whereby the brain minimizes prediction error by adjusting expectations. This would also be compatible with arguments of Frank and Yang (2018), though less with the view of speech tracking expressed in Ding et al. (2016), where speech tracking is seen as reflecting operations of structure building as well.

Our findings are also, especially given the observed premotor TD influence being relevant to WiN, compatible with the framework within which active sensing is a vital mechanism in speech processing and may play a role in determining individual differences in speech processing abilities across individuals (Poeppel and Assaneo 2020).

A further point of interest is the importance of amplitude modulation as a cue in helping listeners to resolve speech in noise. This is reflected in the fact that speakers typically increase the amplitude modulation content of their speech in the presence of background noise (Bosker and Cooke 2018), which hypothetically would serve to enhance the potential for a partially masked signal to drive the SBS that carries “speech rhythm through to comprehension” (Peelle and Davis 2012) and thereby improve chances of accurate speech recognition. Interestingly, the observed relationship between speech envelope trackings in the syllable range (i.e. theta-band) predicts performance on a speech in noise task in which the noise is multi-talker babble, i.e. an amplitude modulated noise with some information content. Although it remains to be seen if this same feature of brain responses in quiet also contributes to performance in stationary noise, we find it reassuring for the potential real-world applicability of these findings, given that much masking noise encountered in everyday life is nonstationary. However, whether this same pattern of inter-individual differences would be found for stationary noise or noise with no informational masking potential remains to be empirically tested. Probing these relationships further in future experiments would provide additional clarity on whether the mechanism at play is one that is relevant to relatively higher-level information segmentation or rather one of “source separation.”

### Comparison to existing literature

A lot of the existing studies dealing with the relationship of SBS and speech perception are studies of elderly populations (Presacco et al. 2016; Goossens et al. 2018; Decruy et al. 2019; Fuglsang et al. 2020; Schmitt et al. 2022). Interestingly, in that literature a consensus appears to have emerged that—at group level—elderly participants exhibit relatively greater speech tracking than their younger counterparts but, at the same time, generally worse speech comprehension performance. At first sight, such reports are at odds with our findings of a positive relationship between the amount of speech tracking and speech-in-noise comprehension. Crucially, however, the results cited above are group-level effects and, as such, must be distinguished from inter-individual effects as reported in the present study. One of the few studies that also examined the relationship between SBS and speech perception performance on an inter-individual level (participant-by-participant) found the same general effect—individuals with larger speech tracking perform better in noisy speech comprehension (Decruy et al. 2019).

Thus, there may be at least two processes or components of speech tracking at play that explain these apparently conflicting results: first, the mean increase in speech-brain coherence found in older—and less well-performing—listeners may be the result of a (central) mechanism that up-modulates sensitivity to the incoming stimuli to compensate for concurrent peripheral degradation (presbycusis). However, this may not automatically result in better perception, but may increase the observed speech-brain coherence. Second, another source of variance may be attributed to a reflection of mechanisms similar to active sensing. This may well result in better speech perception since it reflects an active process that is part of the successful parsing of speech and may be the component of speech tracking that we observed in our study which was predicting WiN performance and also being linked to TD processes. These two mechanisms can act independently of each other or could be even opposing each other and a disentanglement of these two processes would be a next logical step in future work.

### Limitations and outlook

Regarding the question of peripheral vs central factors contributing to the here observed SBS–WiN relationship it has to be noted that no data on the participants’ hearing level such as pure tone audiometry, PTA or similar measures are available. Thus, while we know that the majority of studies reports PTA to be more or less independent of WiN anyway (Blandy and Lutman 2005; Dubno et al. 1984; Duquesnoy 1983; Jerger 1992), it is not possible to conclusively rule out a peripheral origin of the observed relationship between individual differences in SBS and WiN performance. However, connectivity analyses showed that TD directed fronto-temporal connectivity strength partially mediated the observed SBS–WiN relationship. This—and the fact that a converse, bottom-up mediation was not observed—strongly implies that TD effects at least partially drive SBS in quiet, consistent with proposals of SBS (or speech tracking) being an active TD -driven process rather than purely sensory driven.

We believe that the current results further support the notion of cerebral speech tracking reflecting an active mechanism rather than simple passive tracking. It is noteworthy that the individual and intrinsic variation of SBS that we see, despite identical stimuli having been presented to each participant, is unlikely to simply be “noise” or to have a prosaic explanation such as being the result of different head sizes or brain morphology affecting MEG source estimations. Instead, it appears that this intrinsic variation reflects neurophysiologically meaningful differences between individuals. Indeed, such variability in human brain activity, be it spontaneous resting state power or intrinsic connectivity has been repeatedly shown to not only be highly distinctive of individual brains, serving as an individual “fingerprint” (Finn et al. 2015), but also to be functionally relevant, for speech comprehension (Houweling et al. 2020) and cognitive performance in general (Smith et al. 2015; Becker and Hervais-Adelman 2020).

It must be borne in mind that this study has only considered a link between speech tracking and WiN performance on isolated, monosyllabic words. It would be interesting to see whether SBS in quiet is indicative of speech comprehension in more naturalistic adverse conditions and with more complex materials. While such a possibility—a more general role of SBS beyond mono-syllabic words—is not implausible, further studies are needed to test this hypothesis. Another point regarding the use of the different linguistic rates is that alternative approaches such as estimating stress rate rather than using word or syllable rate may be used as predictors of brain activity (e.g. as in Aubanel et al. 2016). The fact that the modulation spectrum shows one relatively wide peak around theta band as opposed to multiple peaks may be seen as another hint that the decomposition into linguistic rates may not be the optimal way of describing the speech signals at hand, but future studies are needed to further elucidate this.

It is worth highlighting that the syllable and phoneme rate encompass frequencies that partially overlap those typically assigned to (spontaneous, task-unrelated) theta and alpha activity. Because of the spectral overlap of the mean rates of the linguistic units and spontaneous brain rhythms, it is reasonable to ask how much of the observed brain activity is the contribution of spontaneous rhythms and how much is the result of the tracking the quasi-rhythmic speech input (i.e. task related or induced). However, the fact that we observe pronounced speech tracking in the theta band frequency or at syllable rate speaks in favor of a meaningful stimulus-driven change in activity, even in the likely presence of underlying task-unrelated theta activity. The absence of strong phoneme-rate tracking provides a less clear picture—it is conceivable, that spontaneous, task-unrelated alpha oscillations may simply mask any concurrent phoneme tracking.

Finally, we note that, the findings reported above remain correlational, and we ultimately cannot determine whether, for example, increased TD connectivity from the areas that we identified causes enhanced SBS, or whether the observed increase in TD connectivity is just an epiphenomenon resulting from enhanced SBS.

We would also like to note that while we have found evidence for an active role of speech tracking, as opposed to a purely passive role, this does not directly touch on the highly debated question about the nature of speech tracking, specifically the debate of entrainment—in the sense of a strict phase resetting of some ongoing, intrinsic rhythm vs the alternative scenario of evoked and additive responses generating the observed brain signals. The field of neurolinguistics (e.g. see Haegens and Golumbic 2018; Obleser and Kayser 2019; Haegens 2020) and in fact neuroscientific fields beyond (e.g. studies about the visual system; Becker et al. 2008; Mazaheri and Jensen 2008) or more general auditory studies (Makeig et al. 2002) are still divided regarding these scenarios, with some researchers favoring entrainment explanations and others favoring evoked responses. The paradigm employed here does not set out to disentangle these two alternatives and cannot do so. However, we would like to emphasize that we, like others (Obleser and Kayser 2019) deem the term “entrainment” in its weaker, or broader, sense less problematic, and this is the way this term is used in this work. Entrainment in the sense of purely showing a phase locking between speech and brain signals can be a useful term, and can be applied without making any (hard to verify or to refute) assumptions about the preexistence of spontaneous rhythms that simply and exclusively reset their phase in response to some incoming stimulus.

In summary, this study provides potential targets to investigate using methods that might allow causal inference, such as noninvasive brain stimulation (TMS, tES), or even connectivity-based M/EEG neurofeedback. These techniques have potential to provide causal evidence for the role of speech tracking and have already shown first success in modulating speech perception or brain responses to speech (Riecke et al. 2018; Wilsch et al. 2018; Zoefel et al. 2018; Keshavarzi et al. 2020; Ruhnau et al. 2020). The fact that we have found subject-by-subject variability of speech tracking to be functionally relevant also speaks to the utility of defining such targets, potentially at an individual level. Our results may help to increase the possible specificity of noninvasive therapeutical approaches—especially the where and the what, i.e. the locus and the frequency of interest for a potential brain stimulation or neurofeedback intervention—and hence impact such an intervention may have.

## Conclusions

The results presented above support the notion that speech tracking reflects a mechanism well beyond passive following of speech input, even in the case of noise-free, clear speech. Inter-individual differences in the magnitude of SBS in quiet have a functional implication, predicting performance in completely independent speech in noise perception tasks. This is adding to our understanding of how speech tracking interacts with perception and cognition and inter-areal communication. By demonstrating TD influences on areas with strong speech tracking it shows that there are cortical processes at play incompatible with simple bottom-up, passive mirroring of the speech envelope. This insight may help toward building better cognitive-cortical models of speech comprehension, from monitoring to intervention, with the ultimate goal of targeting specific brain areas using brain stimulation or neurofeedback. In the future, custom-tailored, cortically mediated intervention may offer a unique therapeutical path forward for individuals that suffer from hearing impairment without a clear sensory-peripheral origin.

## Supplementary Material

MS_CerCortComms_Robert_Becker_SuppMaterial_tgad001

## Data Availability

The raw data originating from HCP can be obtained from HCP directly. Scripts necessary to preprocess and analyze the corresponding HCP data and to reproduce results of this study can be provided on reasonable demand by the authors.
