# Supplementary material for: Individual theta-band cortical entrainment to speech in quiet predicts word-in-noise comprehension
Source: Cereb Cortex Commun. 2023 Jan 5;4(1):tgad001. doi: 10.1093/texcom/tgad001 (PMC9883620; doi:10.1093/texcom/tgad001)
Supplement: MS_CerCortComms_Robert_Becker_SuppMaterial_tgad001 [file ms_cercortcomms_robert_becker_suppmaterial_tgad001.docx]

**Supplementary Material**

Supplementary Table 1. Regions included in parcellation used for estimation of connectivity. Abbreviations: LT = Left-hemispheric CACoh target area, RT = right hemispheric CACoh target area. LFS – left-hemispheric frontal source areas, sending information to left-hemispheric target area LT. RFS – right hemispheric frontal source areas sending information to right-hemispheric target area RT. LPS – left-hemispheric primary auditory area, sending information to left-hemispheric target area LT. RPS – right hemispheric equivalent of LPS. Same areas and connections denoted here are used for the bottom-up case (just inverted). Abbreviations: d = dorsal, dl = dorsolateral, v = ventral, vl = ventrolateral, tl = tongue and larynx region, hf = head and face region, c = caudal, r= rostral, op = part opercularis, m = medial, iv = intermediate ventral.

| Parcel number | Label and Brodmann areas (BA) | Parcel number | Label and Brodmann areas (BA) |
| --- | --- | --- | --- |
| 1 (RFS) | Right Superior Frontal Gyrus (BA6dl), Right Middle Frontal Gyrus (BA6vl), PrG, Right Precentral Gyrus (BA6cdl) | 8 | Left Inferior Frontal Gyrus (BA45r)  Left Inferior Frontal Gyrus (BA44op) |
| 2 (RFS) | Right Inferior Frontal Gyrus (BA45c), right Inferior Frontal Gyrus (BA45r), right Inferior Frontal Gyrus (BA44op) | 9 (LFS) | Left Precentral Gyrus (BA4hf)  Left Precentral Gyrus (BA6cvl) |
| 3 (LFS) | Left Superior Frontal Gyrus (BA6dl), Left Middle Frontal Gyrus (BA6vl), Left Precentral Gyrus (BA6cdl) | 10 (RFS) | Right Precentral Gyrus (BA4hf) |
| 4 (RPS) | Right Superior Temporal Gyrus TE1.0 and TE1.2, Right Precentral Gyrus (BA4tl) | 11 (RFS) | Right Inferior Frontal Sulcus IFS |
| 5 (RFS) | Right Inferior Frontal Gyrus (BA44d), Right Inferior Frontal Gyrus (BA44v), Right Precentral Gyrus (BA6cvl) | 12 (LPS) | Left Superior Temporal Gyrus (TE1.0 and TE1.2) |
| 6 (LFS) | Left Precentral Gyrus (BA4tl) | 13 (RT) | Right Superior Temporal Gyrus, CACoh-ROI |
| 7 (LFS) | Left Inferior Frontal Gyrus (BA44d)  Left Inferior Frontal Gyrus IFS  Left Inferior Frontal Gyrus (BA45c) | 14 (LT) | Left Middle Temporal Gyrus, CACoh-ROI |


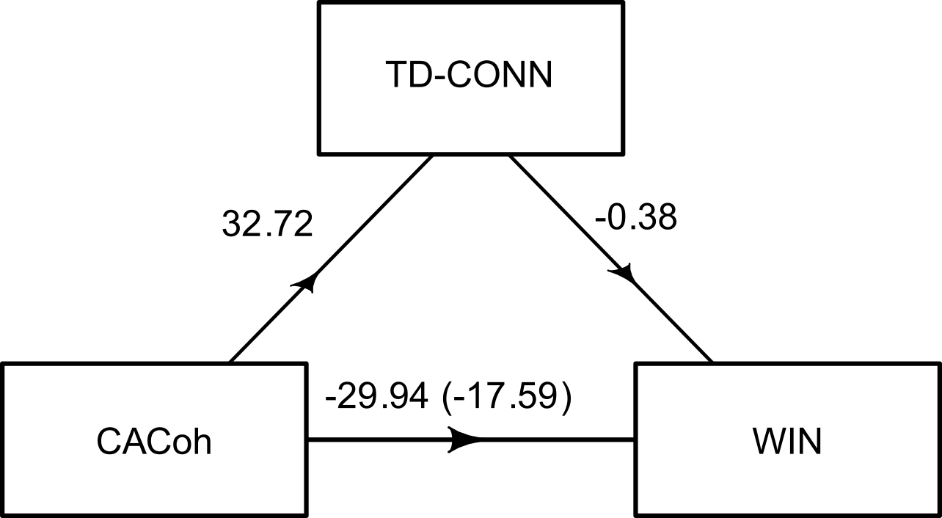


**Supplementary Figure 1.** Mediation analysis. Including CCA-weighted top-down connectivity (TD-CONN) into the model decreased the path coefficient of the direct effect of SBS on WiN by nearly half, from -29.94 (total effect) to -17.59. This, together with the paths a (SBS vs TD) and b (TD-CONN vs WiN) showing significant correlations, a bootstrapping confidence interval outside zero and a trend in the Sobel test (p = 0.059) suggests partial mediation of the observed SBS to WiN relationship by a top-down connectivity, i.e., by a non-peripheral factor.
